# Supplementary material for: Future strengthening of the Nordic Seas overturning circulation
Source: Nat Commun. 2023 Apr 12;14:2065. doi: 10.1038/s41467-023-37846-6 (PMC10097703; doi:10.1038/s41467-023-37846-6)
Supplement: Supplementary file 1 — Supplementary Information [file 41467_2023_37846_MOESM1_ESM.pdf]

Future strengthening of the Nordic Seas overturning circulation

Marius Årthun[1,*], Helene Asbjørnsen[1], Léon Chafik[2,3], Helen L. Johnson[4] & Kjetil Våge[1]

[1]*Geophysical Institute, University of Bergen, and Bjerknes Centre for Climate Research, Bergen, Norway.*

[2]*Department of Meteorology and Bolin Centre for Climate Research, Stockholm University, Stockholm, Sweden*

[3]*National Oceanography Centre, Southampton, UK*

[4]*Department of Earth Sciences, University of Oxford, Oxford, UK.*

**Corresponding author. email: marius.arthun@uib.no*

Supplementary Tables and Figures

In this supporting information we provide additional tables and figures that support the results in the main text.

Supplementary Table 1: CMIP6 models, members, and variables used in the analysis. The ocean nominal horizontal resolution (lon×lat) is also provided. MOC_σ: meridional overturning in density space (model variables *msftmrho* and *msftyrho*). MOC_z: meridional overturning in depth space (model variables *msftmz* and *msftyz*). BSF: barotropic streamfunction (*msftbarot*). SST: sea-surface temperature (*tos*). SSS: sea-surface salinity (*sos*). SHF: net surface heat flux (*hfds*). For BSF, SSP245 and SSP585 were analyzed, while for MOC_σ, SST, SSS and SHF, SSP585 was analyzed.

Model name	Member	Horiz.	$\mathrm{MOC}_{z,\sigma}$	BSF	SST	SSS	SHF	Reference
ACCESS-CM2	r1i1p1f1	1×1	x	x	x	x	x	Ziehn et al. (2020)[1]
ACCESS-ESM1-5	r1i1p1f1	1×1	x	x	x	x	x	Bi et al. (2020)[2]
CanESM5	r1i1p1f1	1×1		x	x	x	x	Swart et al. (2019)[3]
CESM2-WACCM	r1i1p1f1	1×1		x	x	x	x	Danabasoglu et al. (2020)[4]
CIESM	r1i1p1f1	1×1		x	x	x	x	Lin et al. (2020)[5]
CMCC-CM2-SR5	r1i1p1f1	1×1		x	x	x	x	Cherchi et al. (2019)[6]
EC-Earth3	r1i1p1f1	1×1		x	x			Döscher et al. (2022)[7]
FGOALS-g3	r1i1p1f1	1×1		x	x			Li et al. (2020)[8]
GFDL-CM4	r1i1p1f1	0.25×0.25	x		x	x	x	Held et al. (2019)[9]
GFDL-ESM4	r1i1p1f1	0.5×0.5	x		x	x	x	
GISS-E2-1-G	r1i1p1f2	1.25×1		x	x			Kelley et al. (2019)[10]
HadGEM3-GC31-LL	r1i1p1f3	1×1		x	x	x	x	Kuhlbrodt et al. (2018)[11]
IPSL-CM6A-LR	r1i1p1f1	1×1		x	x	x	x	Lurton et al. (2020)[12]
MIROC6	r1i1p1f1	1×1		x	x			Tatebe et al. (2019)[13]
MPI-ESM1-2-LR	r1i1p1f1	1.5×1.5		x	x	x	x	Mauritsen et al. (2019)[14]
MPI-ESM1-2-HR	r1i1p1f1	0.4×0.4		x	x	x	x	Müller et al. (2018)[15]
MRI-ESM2-0	r1i1p1f1	1×0.5		x	x	x	x	Yukimoto et al. (2019)[16]
NorESM2-LM	r1i1p1f1	1×1	x	x	x	x	x	Seland et al. (2020)[17]
NorESM2-MM	r1i1p1f1	1×1	x		x	x	x	
UKESM1-0-LL	r1i1p1f2	1×1		x	x	x	x	Sellar et al. (2020)[18]

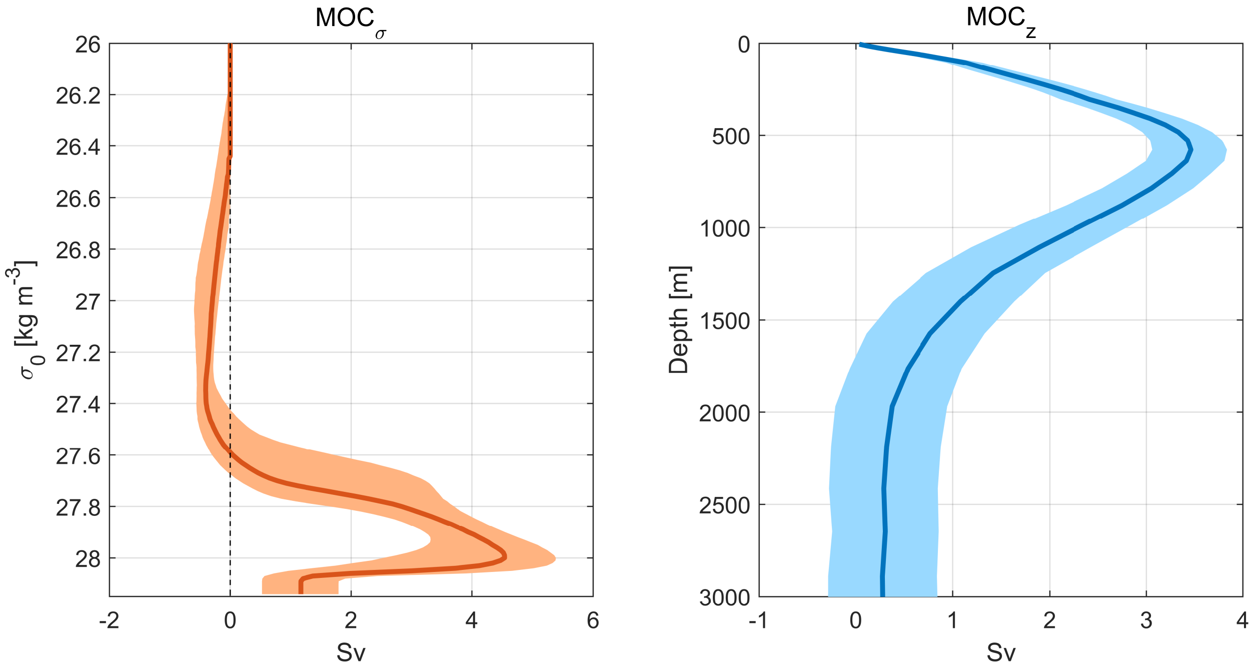

Supplementary Figure 1: Mean overturning streamfunctions (1920-2100) in CESM-LE at 70°N in (left) density-space and (right) depth-space. Solid line shows the ensemble mean and the shading indicates the interquartile spread across ensemble members and time.

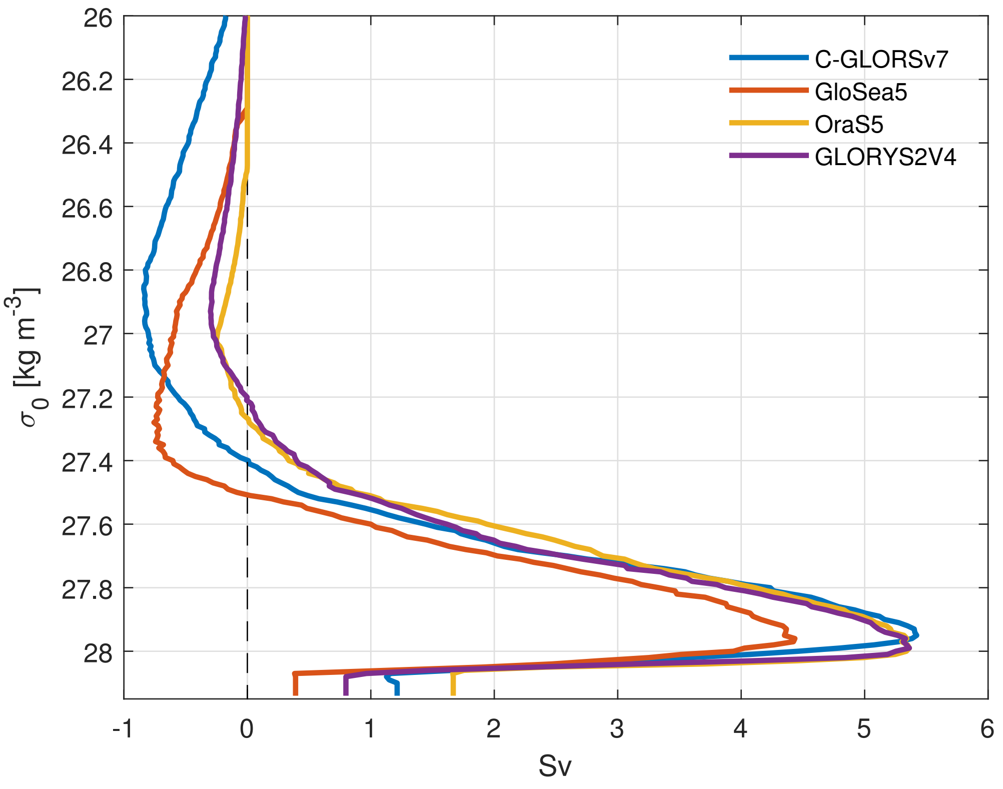

Supplementary Figure 2: Mean overturning streamfunctions for four ocean reanalyses between 2010 and 2019.

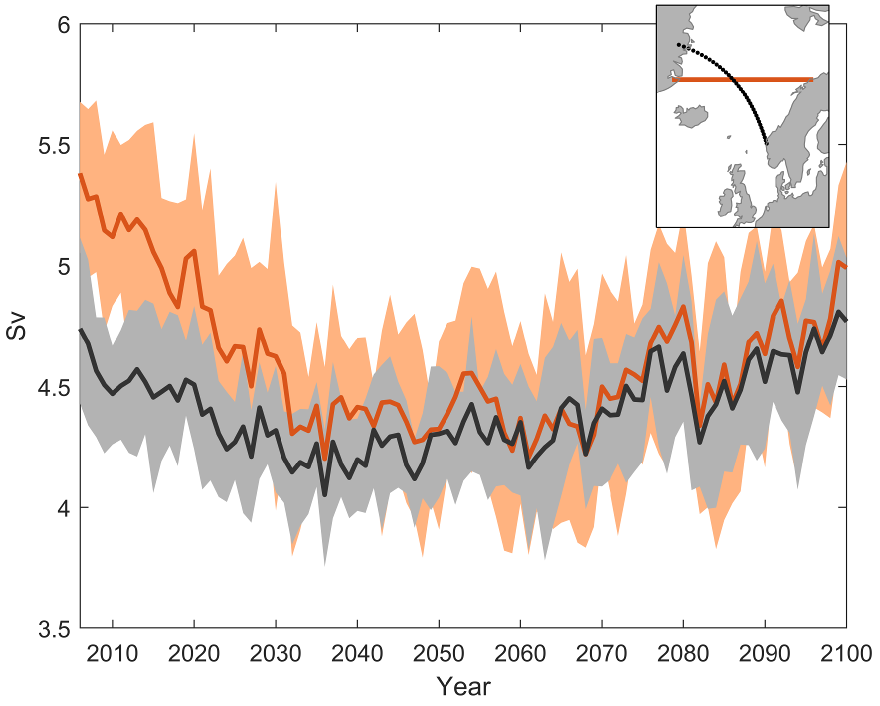

Supplementary Figure 3: Time series of maximum overturning strength in density space in CESM-LE at 70°N (red line) and for a section crossing the Nordic Seas on the native model grid (black line). Solid lines show the ensemble mean and the shading indicates the interquartile spread. Sections are shown on the inset map.

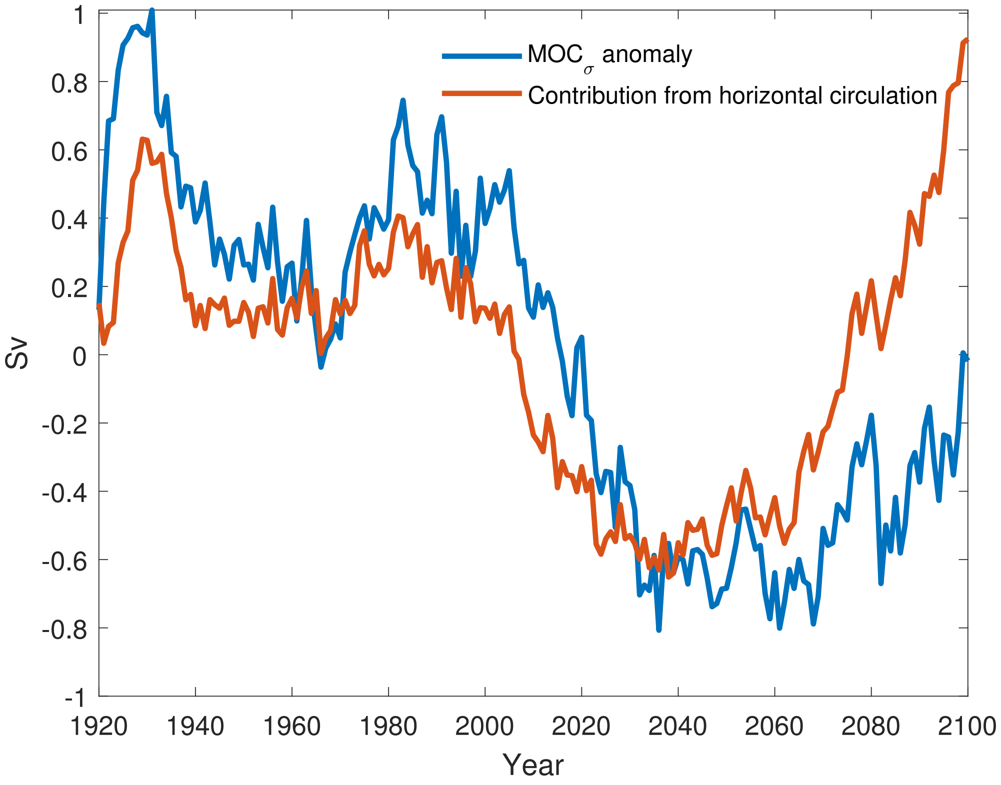

Supplementary Figure 4: Contribution of horizontal circulation to the Nordic Seas overturning streamfunction. Blue line shows the anomalous overturning circulation (relative to the long-term mean), while the red line shows the difference between the overturning circulation in density-space and depth-space. The latter approximates the contribution of horizontal circulation[19].

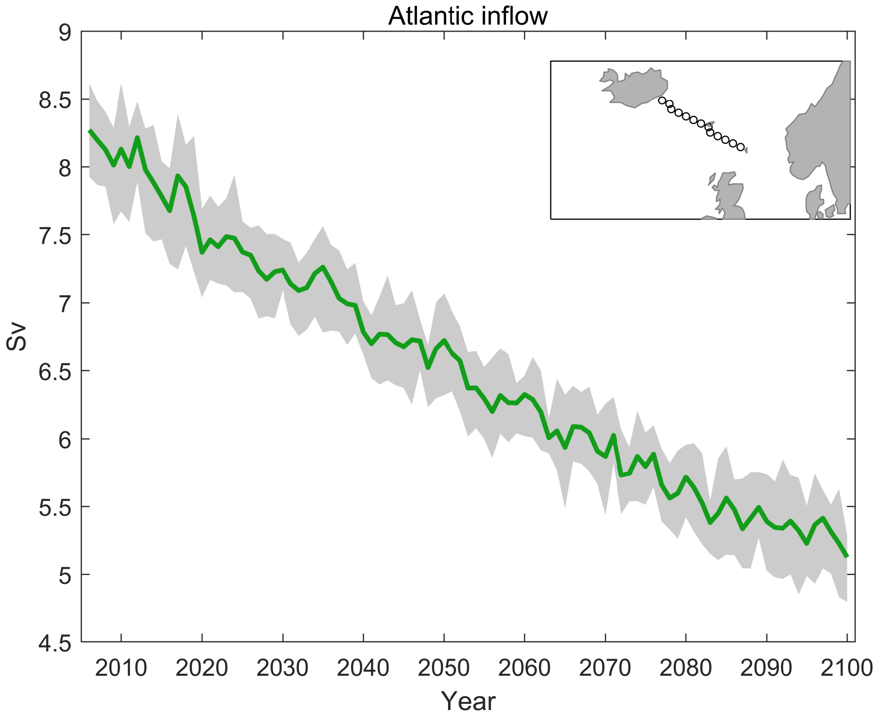

Supplementary Figure 5: Inflow (volume transport) between Iceland and the Faroe Islands in CESM-LE. Solid line shows the ensemble mean and the shading indicates the interquartile spread. The section is shown in the inset map.

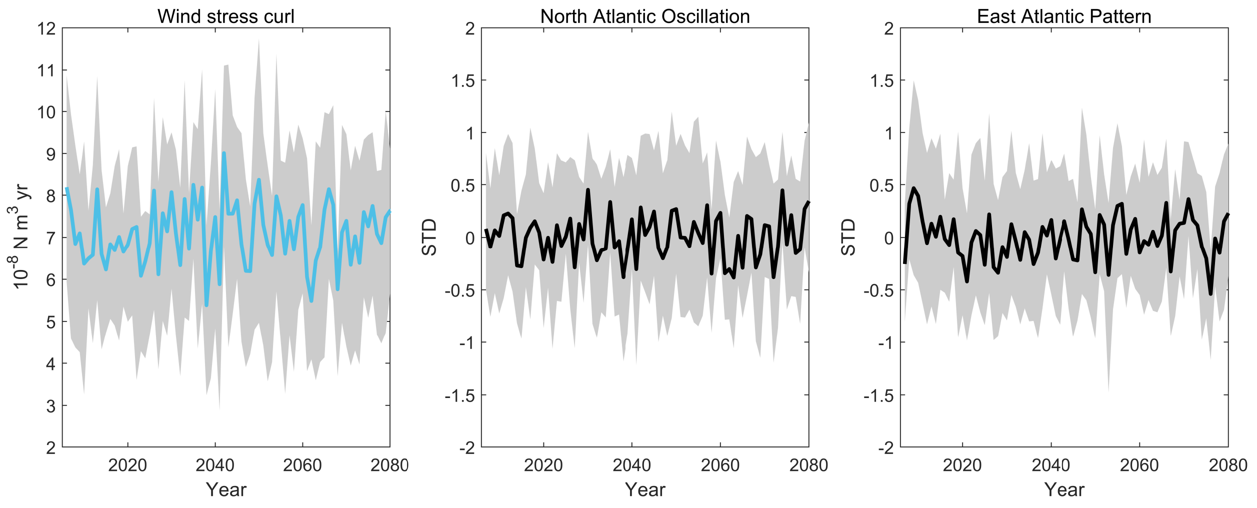

Supplementary Figure 6: Time series of (a) wind stress curl over the Nordic Seas (63-76°N, 10°W-10°E), (b) the North Atlantic Oscillation index, and (c) the East Atlantic Pattern index in CESM-LE. Solid line shows the ensemble mean and the shading indicates the interquartile spread.

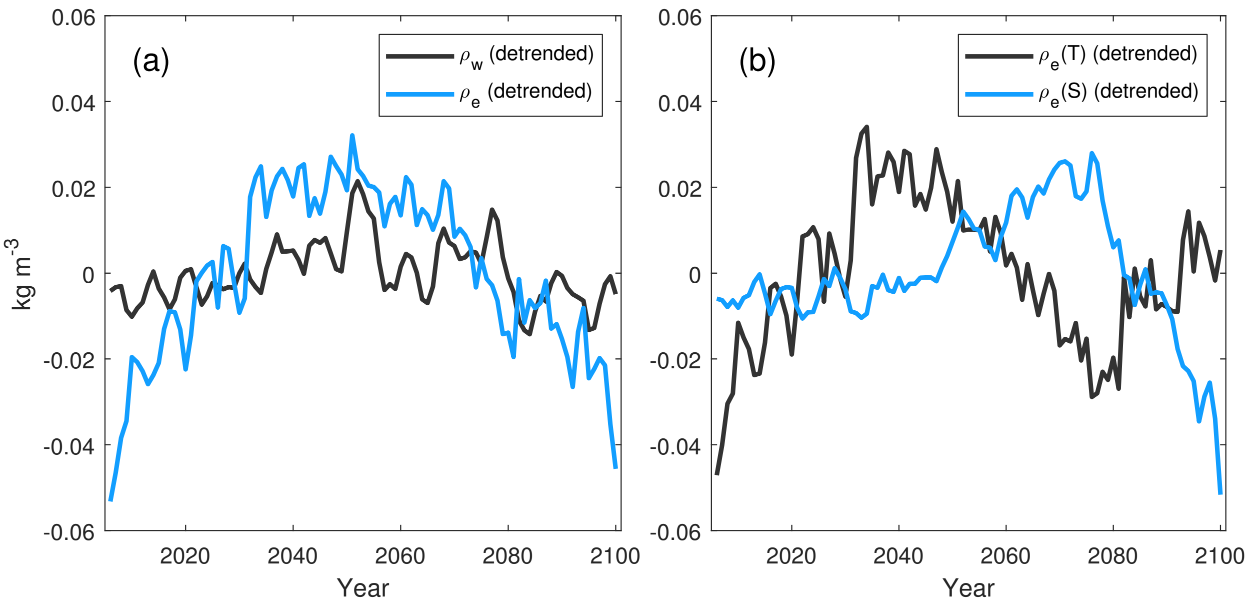

Supplementary Figure 7: (a) Time series of detrended density (200-500m) at the western and eastern boundary, $\rho_{w,e}$ of the Nordic Seas at 70°N. (b) Contributions from temperature (T) and salinity (S) to density changes at the eastern boundary (ρ_e).

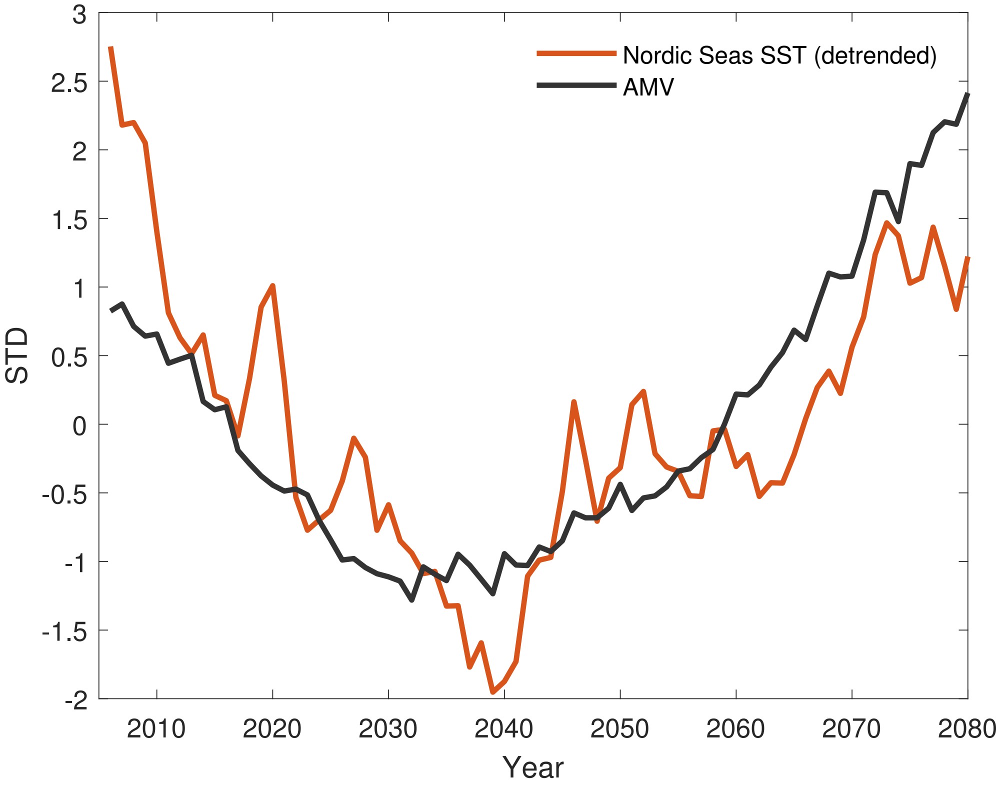

Supplementary Figure 8: Time series of detrended SST in the northern Nordic Seas (70-80°N) and the Atlantic Multidecadal Variability (AMV) index. The AMV index is calculated from low-frequency component analysis[20,21] (see Methods in main manuscript for further details).

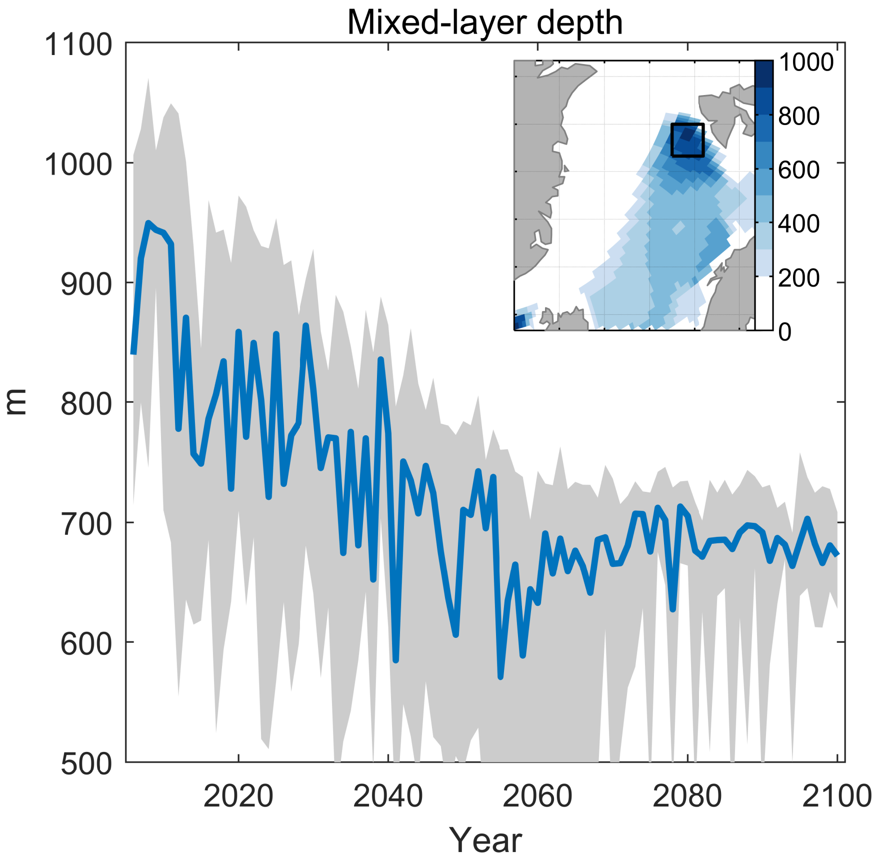

Supplementary Figure 9: Time series of mixed-layer depth (MLD) in March in the region of deep convection in the northern Nordic Seas (76-78°N, 5-12°E). The inset map shows the mean MLD between 2011-2020 and the black box shows the region used to calculate the time series.

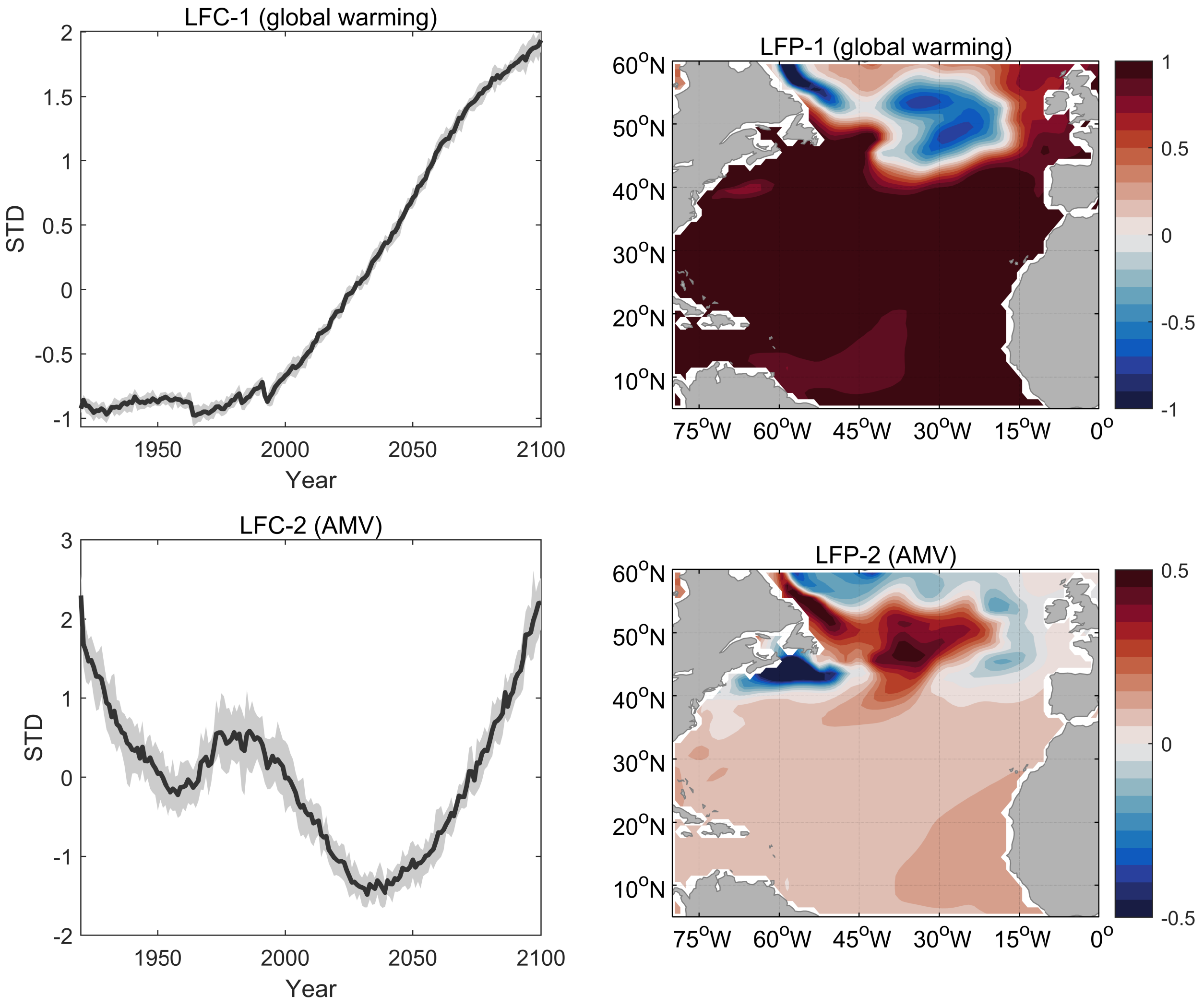

Supplementary Figure 10: Time series and spatial patterns of the first two modes of low-frequency variability in CESM-LE.

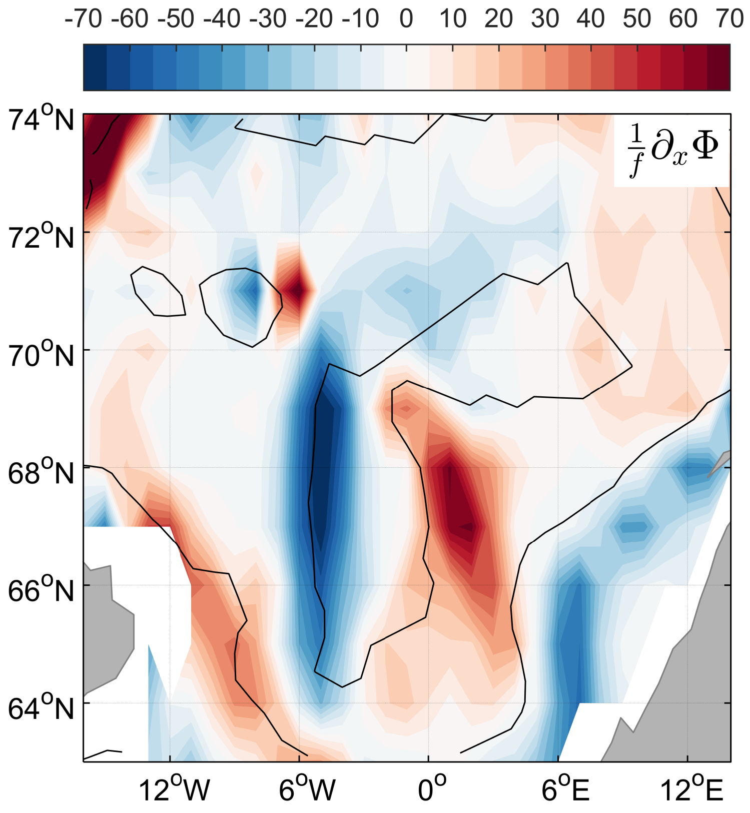

Supplementary Figure 11: Potential energy term calculated as in REF. 22 where $\Phi = g\int_{-H}^{0}\Delta\rho z dz$; $\Delta\rho = \left[(\rho(34.9, -1, z) - \rho(S, T, z)\right] / \rho(34.9, -1, 0)$.

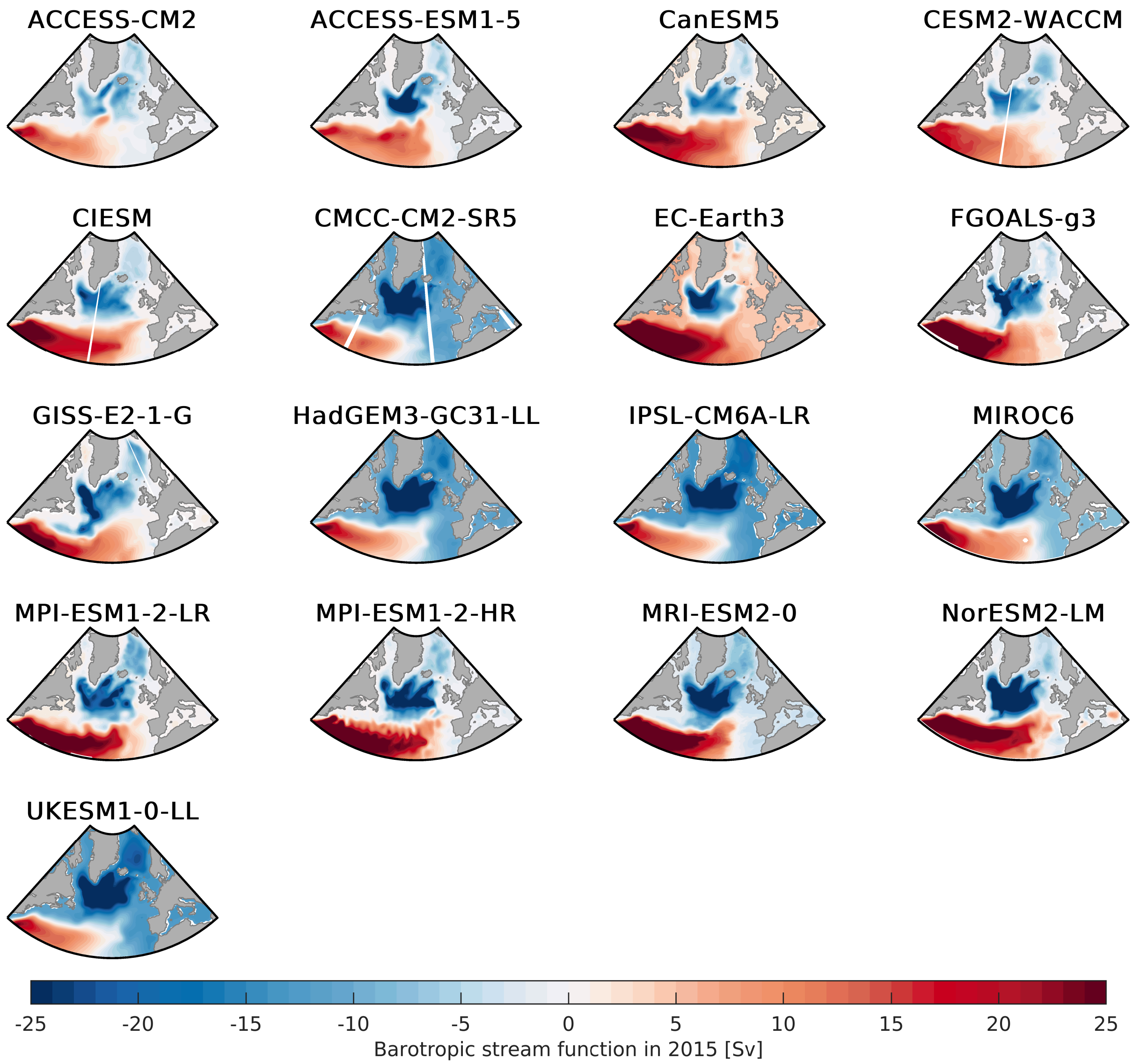

Supplementary Figure 12: Barotropic streamfunction in 2015 for CMIP6 models used in this study.

Supplementary References

1. Ziehn, T. *et al.* The Australian earth system model: ACCESS-ESM1. 5. *Journal of Southern Hemisphere Earth Systems Science* **70**, 193–214 (2020).

2. Bi, D. *et al.* Configuration and spin-up of ACCESS-CM2, the new generation Australian community climate and earth system simulator coupled model. *Journal of Southern Hemisphere Earth Systems Science* **70**, 225–251 (2020).

3. Swart, N. C. *et al.* The Canadian earth system model version 5 (CanESM5. 0.3). *Geoscientific Model Development* **12**, 4823–4873 (2019).

4. Danabasoglu, G. *et al.* The community earth system model version 2 (CESM2). *Journal of Advances in Modeling Earth Systems* **12**, e2019MS001916 (2020).

5. Lin, Y. *et al.* Community integrated earth system model (CIESM): Description and evaluation. *Journal of Advances in Modeling Earth Systems* **12**, e2019MS002036 (2020).

6. Cherchi, A. *et al.* Global mean climate and main patterns of variability in the CMCC-CM2 coupled model. *Journal of Advances in Modeling Earth Systems* **11**, 185–209 (2019).

7. Döscher, R. *et al.* The EC-Earth3 Earth system model for the Coupled Model Intercomparison Project 6. *Geoscientific Model Development* **15**, 2973–3020 (2022).

8. Li, L. *et al.* The flexible global ocean-atmosphere-land system model grid-point version 3 (FGOALS-g3): description and evaluation. *Journal of Advances in Modeling Earth Systems* **12**, e2019MS002012 (2020).

9. Held, I. *et al.* Structure and performance of GFDL's CM4. 0 climate model. *Journal of Advances in Modeling Earth Systems* **11**, 3691–3727 (2019).

10. Kelley, M. *et al.* GISS-E2. 1: Configurations and climatology. *Journal of Advances in Modeling Earth Systems* **12**, e2019MS002025 (2020).

11. Kuhlbrodt, T. *et al.* The low-resolution version of HadGEM3 GC3. 1: Development and evaluation for global climate. *Journal of Advances in Modeling Earth Systems* **10**, 2865–2888 (2018).

12. Lurton, T. *et al.* Implementation of the CMIP6 Forcing Data in the IPSL-CM6A-LR Model. *Journal of Advances in Modeling Earth Systems* **12**, e2019MS001940 (2020).

13. Tatebe, H. *et al.* Description and basic evaluation of simulated mean state, internal variability, and climate sensitivity in MIROC6. *Geoscientific Model Development* **12**, 2727–2765 (2019).

14. Mauritsen, T. *et al.* Developments in the MPI-M Earth System Model version 1.2 (MPI-ESM1. 2) and its response to increasing CO2. *Journal of Advances in Modeling Earth Systems* **11**, 998–1038 (2019).

15. Müller, W. A. *et al.* A higher-resolution version of the max planck institute earth system model (MPI-ESM1. 2-HR). *Journal of Advances in Modeling Earth Systems* **10**, 1383–1413 (2018).

16. Yukimoto, S. *et al.* The Meteorological Research Institute Earth System Model version 2.0, MRI-ESM2. 0: Description and basic evaluation of the physical component. *Journal of the Meteorological Society of Japan. Ser. II* (2019).

17. Seland, Ø. *et al.* Overview of the Norwegian Earth System Model (NorESM2) and key climate response of CMIP6 DECK, historical, and scenario simulations. *Geoscientific Model Development* **13**, 6165–6200 (2020).

18. Sellar, A. A. *et al.* Implementation of UK Earth system models for CMIP6. *Journal of Advances in Modeling Earth Systems* **12**, e2019MS001946 (2020).

19. Zhang, R. & Thomas, M. Horizontal circulation across density surfaces contributes substantially to the long-term mean northern Atlantic Meridional Overturning Circulation. *Communications Earth & Environment* **2**, 1–12 (2021).

20. Wills, R. C., Armour, K. C., Battisti, D. S. & Hartmann, D. L. Ocean–atmosphere dynamical coupling fundamental to the Atlantic multidecadal oscillation. *Journal of Climate* **32**, 251–272 (2019).

21. Årthun, M., Wills, R. C., Johnson, H. L., Chafik, L. & Langehaug, H. R. Mechanisms of decadal North Atlantic climate variability and implications for the recent cold anomaly. *Journal of Climate* **34**, 3421–3439 (2021).

22. Broomé, S., Chafik, L. & Nilsson, J. Mechanisms of decadal changes in sea surface height and heat content in the eastern Nordic Seas. *Ocean Science* **16**, 715–728 (2020).
